# Direct evidence of correlation between the second harmonic generation anisotropy patterns and the polarization orientation of perovskite ferroelectric

**DOI:** 10.1038/s41598-017-09339-2

**Published:** 2017-08-22

**Authors:** Jie-su Wang, Kui-juan Jin, Jun-xing Gu, Qian Wan, Hong-bao Yao, Guo-zhen Yang

**Affiliations:** 1 Beijing National Laboratory for Condensed Matter Physics, Institute of Physics, Chinese Academy of Sciences, Beijing, 100190 China; 2 University of Chinese Academy of Sciences, Beijing, 100049 China; 3 Collaborative Innovation Center of Quantum Matter, Beijing, 100190 China

## Abstract

For ferroelectric materials, where the polar state breaks the inversion symmetry, second harmonic generation is a useful tool to prove their ferroelectric properties. However, the correlation between the anisotropy patterns and the polarization orientation of the ferroelectric domains has not been clarified yet. In this work, we systematically investigated this correlation in a typical perovskite oxide ferroelectric, Barium Titanate (BaTiO_3_) crystal, by second harmonic generation and the piezoresponse force microscopy technique. The evolution of polarization-dependent anisotropy patterns proves that there is a linear relationship between the rotation angle of second harmonic generation anisotropy patterns and the polarization angle of BaTiO_3_ single crystals. It is a direct evidence illustrating that the polarization of BaTiO_3_ crystal can be qualitatively identified in 0°–180° by second harmonic generation technology. This work gives a glance at improving a nonintrusive and convenient method to identify the polarization of perovskite ferroelectric materials.

## Introduction

Perovskite oxide ferroelectrics have attracted great interests as a candidate class of materials used in visible-light-absorbing and photovoltaic devices^1^. Probing their ferroelectric properties non-destructively is crucial for further both electrical and optical tests, yet there is none well-established technique hitherto. The most promising method to do so should be optical second harmonic generation (SHG)^2–4^, which is one of the nonlinear processes occurring in non-centrosymmetric crystals via the second-order nonlinear susceptibility *χ* ^(2)^ that relates the induced second-order polarization with the applied fundamental optical field^5^. As a noninvasive technique, SHG can be applied under various conditions, such as magnetic or electric field, high-temperature or hypothermal environment and pressure^3, 6, 7^. Since it is highly associated with the symmetry of the object, SHG can give a visual information on different structures with the special resolution of hundred microns and down to 1 micron, mainly depending on whether involving objective lens or not. Based on the high sensitivity of SHG on symmetry changing, second-harmonic microscopy system has been developed and employed in many impressing explorations in physics and biology, such as nonlinear edge resonance of MoS_2_ monolayer^8^, domain structures in thin films^9^, and quantitative analysis of collagen fibrillar structure^10^.

The mostly used SHG technology to verify the symmetry of a sample is the SHG anisotropy pattern, which is also called SHG polarization diagram. Previous researches have mainly related its variation to structural changing and even determined domain structures and symmetries of samples by mapping their SHG anisotropy patterns^3, 4, 11^. A set of papers from the group of Venkatraman Gopalan have used SHG to reveal the domain variety of perovskite ferroelectrics and their nonlinear properties^2, 9, 12, 13^. Other researchers, such as H. Yokota and Morgan Trassin *et al*., have proved the shapes of SHG anisotropy patterns are different in various polarization directions of CaTiO_3_ and BiFeO_3_ samples^3, 14^. However, the correlation between SHG anisotropy patterns and the polarization orientation of ferroelectric domains has not been systematically clarified yet, which hinders SHG to be a more convincing and reliable technique in sufficiently illustrating the ferroelectric information of solid state materials.

Barium Titanate (BaTiO_3_, BTO) has outstanding ferroelectric and piezoelectric properties at room temperature, thus has continuously attracted much attention for its potentially applications in nonvolatile memory storage devices, optical switches, high-density capacitors and electro-optical devices^15, 16^. As BaTiO_3_ is a carefully studied ferroelectric material, which has been widely used as a model system for the explanation of ferroelectric phenomena^17^, in this paper we explore the azimuth angle-resolved SHG anisotropy patterns of this classic ferroelectric material, tetragonal BTO perovskite single crystal^18, 19^, and illuminate their evolution. The samples measured are 10 × 10 × 0.5 mm^3^ (100)-cut BTO crystals with tetragonal symmetry in room temperature and ferroelectric curie temperature *T*_*C*_ = 125 °C^20^. The band width of crystal BTO in room temperature is around 3.2 eV, which means there is no absorption for light at 800 nm or 400 nm. Together with determining the polarization direction of the samples by piezoresponse force microscopy (PFM), it is demonstrated that there is a linear relationship between the rotating angle of SHG anisotropy pattern and the net polarization orientation of a ferroelectric material.

## Results

**Figure 1:**
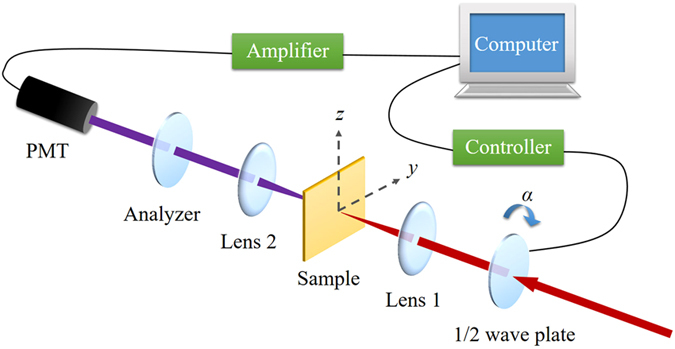
The experimental schematic for SHG transmission measurement. *α* stands for the angle between directions of the incident light polarization and the *y*-axis, which is modulated by a half-wave (*λ*/2) plate assembled on a stepping motor controlled by a computer. Lens 1 is the focusing lens, while lens 2 is for collimating. The laboratory coordinate system is established as shown which is not sample dependent.

**Figure 2:**
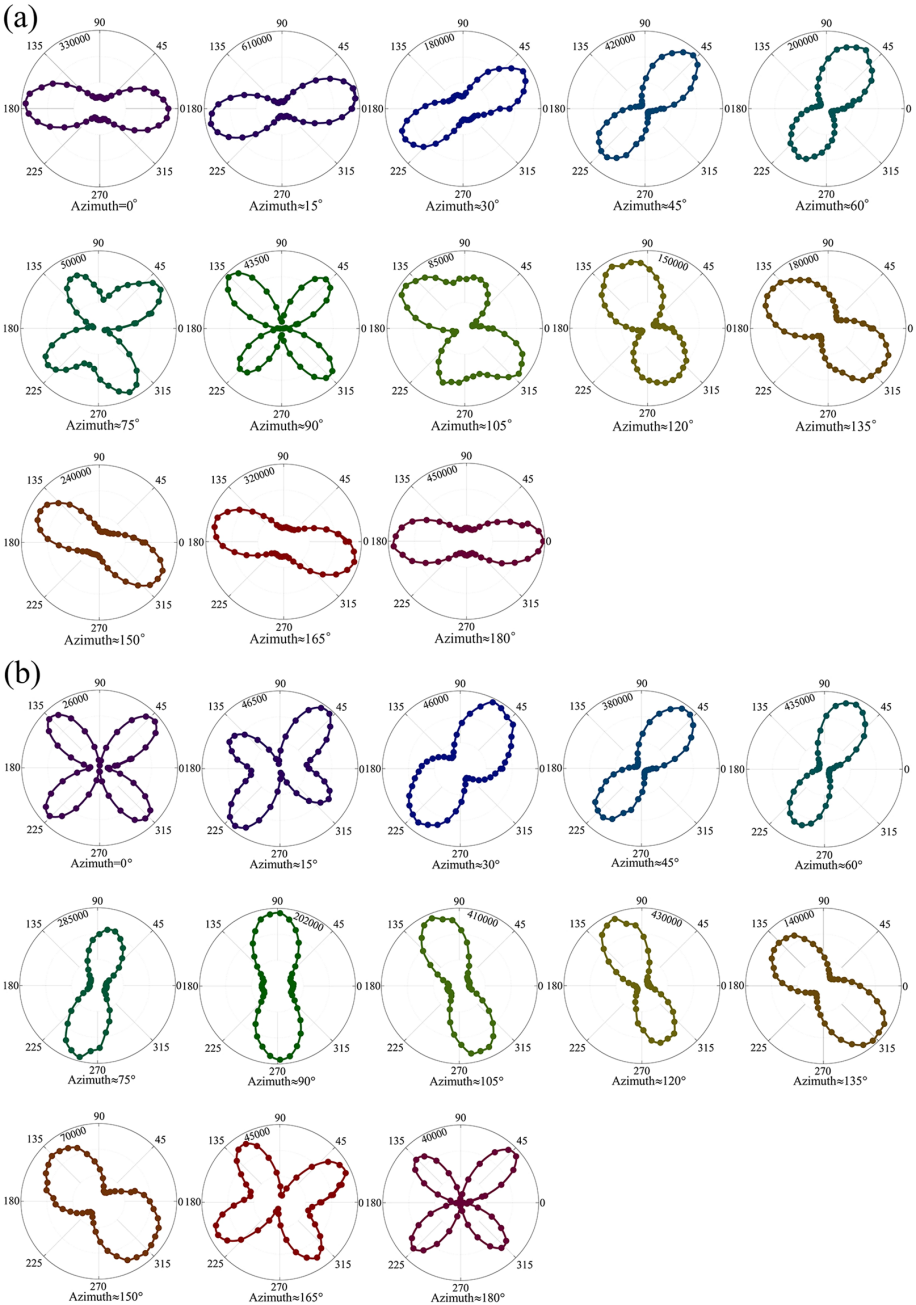
SHG anisotropy patterns in different azimuth angles under (**a**) perpendicular and (**b**) parallel configurations. The colored circles are experimental data, and they are linked to explicitly show the shapes of the SHG patterns.

The SHG anisotropy patterns are collected by a typical SHG transmission setup as shown in Fig. 1. To ensure the incident light focusing on the same area while rotating samples, image magnification system and multidimensional translation stage are engaged to monitor and adjust the position of the focused light spot. Figure 2 shows the results of SHG anisotropy patterns under perpendicular and parallel configurations^21^ (see Methods). It can be clearly seen that these patterns evolve with azimuth changing, where azimuth is the rotation angle of sample relative to its initial position.

As can be seen in Fig. 2a, under the perpendicular configuration, when azimuth changes from 0° to 90°, the patterns evolve from having a twofold rotational symmetry to four-valve structure with the major axis rotating and the maximum SHG intensities decreasing simultaneously. The shapes of patterns in azimuth = 0°, 15°, and 30° are long and narrow. While for azimuth = 45° and 60°, the shapes become much wider with smaller SHG intensity. The peaks begin to split and clear protuberances can be seen in the pattern for azimuth = 75°. Eventually, when azimuth = 90°, each main peak splits into two comparable peaks and the SHG intensity drops to the minimum value with one order of magnitude decrease comparing with that in azimuth = 0°. For the azimuth from 90° to 180°, the whole structure remains rotating accompanying with the separated peaks rejoining into one, meanwhile the SHG intensity has recovered. The patterns with the azimuth in 180°–360° possess the same evolutional features as those with azimuth evolving from 0° to 180° (not shown here).

As to parallel configuration, shown in Fig. 2b, the patterns evolve from having four-valve structure for the initial pattern to having two predominate peaks for azimuth = 90°, and eventually re-separate into four comparable peaks for azimuth = 180°. The evolution of the patterns for the next 180° repeats the process (not shown here). Figure 2a and b prove that the azimuth-dependent SHG anisotropy patterns is a gradual changing process with the fluctuation of SHG intensity.

**Figure 3:**
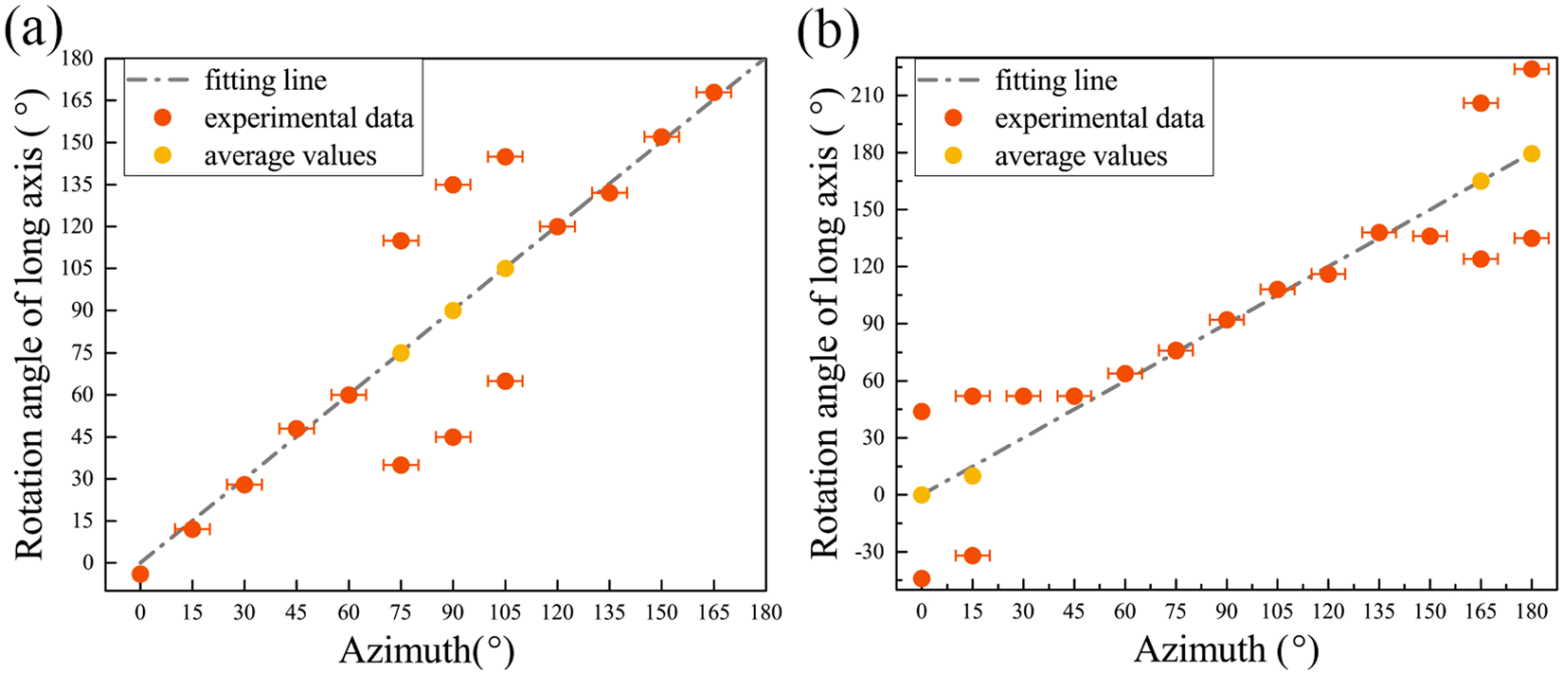
Azimuth angle dependences of the major axes of SHG patterns under (**a**) perpendicular and (**b**) parallel configurations. Error bars in these figures are from changing the sample azimuth angle manually.

In order to illustrate the correlation more directly, the dependence of the rotation angle of major axis in the patterns upon the azimuth of samples is shown in Fig. 3. For the pattern which has two comparable axes, the average angle of the two axes is plotted. The grey dash dot lines are the fittings to experimental data using the formula “*y = x*”. Obviously, the line and the experimental data fit very well, indicating that there is a linear correlation between the patterns and the azimuths. Since the incident light is along sample normal, the non-centrosymmetry out-plane contributes little to the second harmonic (SH) signal. Namely, the SH signal mostly generated from the net polarization in-plane, which rotates synchronously with the samples. Thus, it proves that the polarization orientation of samples has a direct and linear relationship with the SHG anisotropy pattern.

As mentioned above, the SH signals are mostly generated from the polarization in-plane. For (100)-cut tetragonal BTO crystals (point group 4 mm)^5, 16, 22^, it means that the crystal axis *z* is in *y-z* plane of laboratory coordinate shown in Fig. 1. Based on the SHG model, we theoretically analyzed and simulated the experimental results (see Methods). *θ* is defined as the angle between the *z* axis of crystal and laboratory coordinate. Please note that the *θ* here is *not* the same definition as the azimuth mentioned before. From the theoretical analysis, it can be derived that the SHG anisotropy pattern would have a four-valve structure in the cases that *θ* = 0° and 180° for perpendicular and *θ* = 90° and 270° for parallel configuration. Therefore, it can be further concluded that the SHG anisotropy pattern with fourfold rotational symmetry would be obtained in the cases when the net polarization of samples is perpendicular to the polarization direction of analyzer.

**Figure 4:**
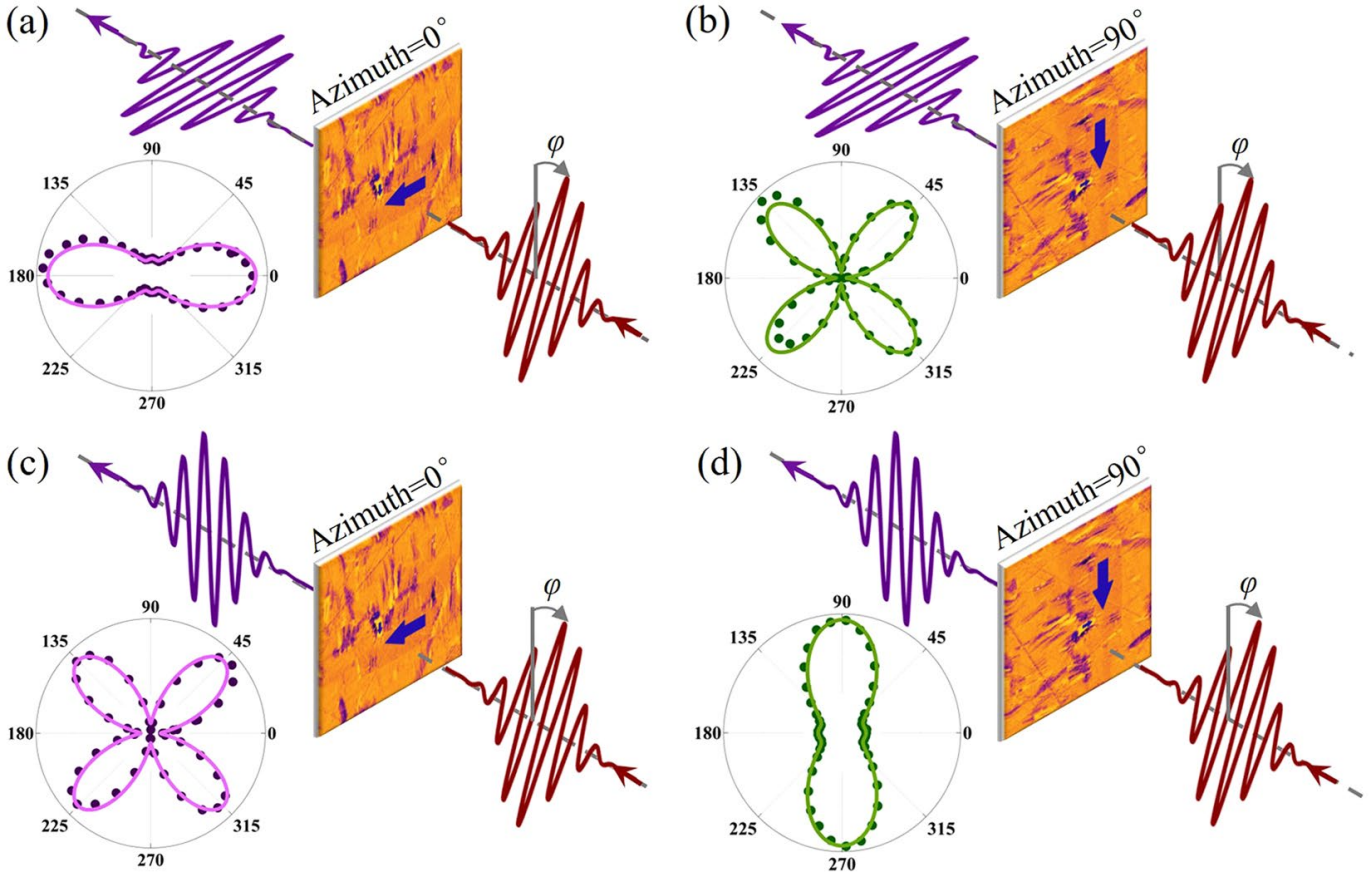
Visual correlation between SHG anisotropy patterns and the dominated polarization orientations (blue arrow) in perpendicular configuration at (**a**) azimuth = 0° and (**b**) 90° and in parallel configuration at (**c**) azimuth = 0° and (**b**) 90°. The polarization of optical fields after analyzer and 1/2 wave plate are colored in purple and red respectively. In four polar plots, the colored circles show the experimental data, while the simulation results are plotted with solid lines.﻿*φ* = ﻿﻿*α*﻿﻿.

Consequently, we deduce that the net polarization of the measured BTO sample is along *y-*axis at the initial position, namely, azimuth = 0°, *θ* = 90°. To further prove this, in-plane PFM scan is engaged to characterize the ferroelectric polarization characteristic of the sample. As shown in Fig. 4, the measured area is 35 μm × 35 μm in the light spot where generates SH signal. The PFM scan in Fig. 4a reveals that, at the initial position, the polarization feature of the sample is exactly dominated by the domain whose polarization is along *y*-axis, which proves the correctness of our deduction. We also investigated the PFM responses of several other areas around in the light spot and found they are all consistent, with the main polarization along *y-*axis.

Figure 4a–d visually illustrate the correlation between SHG anisotropy pattern and the dominated polarization orientation (blue arrow) at azimuth = 0° and 90° in both configurations. One can see clearly that once the net polarization of BTO and the polarization direction of analyzer are parallel (Fig. 4a and d), the obtained SHG anisotropy pattern is featured by the twofold rotational symmetry with a pair of major valves, and once they are perpendicular to each other, the pattern can be distinguished by the fourfold rotational symmetry with four valves (Fig. 4b and c). These results are consistent with our former works^23^. The solid lines in polar plots in Fig. 4 are theoretical simulations with the formula reckoned in the Methods section. For perpendicular configuration, shown as Fig. 4a and b, the theoretical simulations are carried out with *θ* = 90° and 0° respectively. For parallel configuration, shown as Fig. 4c and d, the theoretical simulations are also carried out with *θ* = 90° and 0° respectively. As can be seen, the theoretical results have a good agreement to the experimental data.

Along with the anisotropy pattern evolution in Fig. 2, we can conclude that the polarization of BTO crystal can be qualitatively identified in 0°–180° by SHG technology. It should be noticed that the minimum SHG signal is not zero, which could be resulted from an overlapping of SHG signals generated from other domains in the light spot area.

## Discussion

In this work, direct evidence of correlation between SHG anisotropy patterns and the polarization orientations of BTO single crystals has been systematically investigated by SHG and PFM techniques. The evolution of polarization-dependent anisotropy patterns proves that there is a linear relationship between the rotation angle of the SHG anisotropy patterns and the polarization angle of BTO single crystals. Since theoretically the intensity of polarization is closely related to SHG intensity, this work indicates that SHG can be a promising technique to exactly probe both the orientation and the quantification of net polarization for the perovskite ferroelectric materials. Further application approaching some devices for detecting the polarization orientations of materials based on the present results is highly expected.

## Methods

### Second-Harmonic Generation (SHG) measurement

We got the SHG anisotropy patterns with a typical SHG transmission setup as shown in Fig. 1. The incident laser is generated from a Ti:Sapphire oscillator with central wavelength at 800 nm, pulse duration of 120 fs and repetition of 82 MHz. The energy of incident light was attenuated to 3 mW before being focused. The laboratory coordinate is chosen by directing light propagating along its *–x* direction. The initial polarization of the incident light is set along *z* direction and rotated by a 1/2 wave plate. Single photon counting technique is conducted to count second-harmonic photons, indicating the intensity of second-harmonic signal generated from samples. An image magnification system and multidimensional translation stage are engaged to ensure the incident light focuses on the same area while rotating samples, which guarantees that the net polarization orientation of light spot rotates synchronously with the sample azimuth. Because of the configuration we employed, which includes strong light filed and up to 0.5 mm sample thickness in the straight light path, we believe that the photorefractive effect in BTO crystals contributes little to the measured SH signal.

### In-plane piezoresponse force microscopy (PFM) measurement

The PFM is on a commercial atomic force microscope (AFM, Asylum Research MFP-3D) and the PFM images were collected and recorded using a Ti/Ir-coated Si cantilever (Olympus Electrilever) with a nominal ~2 N/m spring constant and a free air resonance frequency of ~73 kHz. The size of measured area shown in this paper as Fig. 4 is 35 μm × 35 μm and this area is covered by the focused light spot.

### SHG theoretical analysis

For a crystal BTO in its tetragonal phase (point group 4 mm)^22^, the optical second-order susceptibility tensor has a form of ^5, 16^.

1 $${\overleftrightarrow{{\chi }}}_{{4mm}}^{({2})}=(\begin{array}{cccccc}{0} & {0} & {0} & {0} & {{\chi }}_{{15}} & {0}\\ {0} & {0} & {0} & {{\chi }}_{{15}} & {0} & {0}\\ {{\chi }}_{{31}} & {{\chi }}_{{31}} & {{\chi }}_{{33}} & {0} & {0} & {0}\end{array}).$$

We define *θ* as the angle between the *z* axis of crystal and laboratory coordinate. Using coordinate transformation, the effective optical second-order susceptibility tensor under the laboratory coordinate takes the form of

2 $$\begin{array}{c}{\overleftrightarrow{{\chi }}}_{{lab}}^{{(}2{)}}=(\begin{array}{ccc}{1} & {0} & {0}\\ {0} & \cos \,{\theta } & \sin \,{\theta }\\ {0} & {-}\,\sin \,{\theta } & \cos \,{\theta }\end{array})(\begin{array}{cccccc}{0} & {0} & {0} & {0} & {{\chi }}_{{15}} & {0}\\ {0} & {0} & {0} & {{\chi }}_{{15}} & {0} & {0}\\ {{\chi }}_{{31}} & {{\chi }}_{{31}} & {{\chi }}_{{33}} & {0} & {0} & {0}\end{array})\\ \quad \quad =(\begin{array}{cccccc}{0} & {0} & {0} & {{\chi }}_{{15}} & {0} & {0}\\ {{\chi }}_{{31}}\,\sin \,{\theta } & {{\chi }}_{{31}}\,\sin \,{\theta } & {{\chi }}_{{33}}\,\sin \,{\theta } & {{\chi }}_{{15}}\,\cos \,{\theta } & {0} & {0}\\ {{\chi }}_{{31}}\,\cos \,{\theta } & {{\chi }}_{{31}}\,\cos \,{\theta } & {{\chi }}_{{33}}\,\cos \,{\theta } & {-}{{\chi }}_{{15}}\,\sin \,{\theta } & {0} & {0}\end{array})\end{array}$$

Substituting equation (2) into the SH signal intensity expression and according to the relation between photon and light intensity^4, 5^, the sum of photons *S* detected in the two configurations are

3 $$\{\begin{array}{c}{S}_{\perp }\propto {|{\vec{e}}_{2\omega }^{\text{'}}\cdot {\overleftrightarrow{\chi }}_{lab}^{(2)}:{\vec{e}}_{\omega }^{\text{'}}{\vec{e}}_{\omega }^{\text{'}}|}^{{2}}\\ \quad =\,{[{L}_{yy}^{2\omega }{({L}_{yy}^{\omega })}^{{2}}{\chi }_{31}\sin \theta {\sin }^{{2}}\alpha +{L}_{yy}^{{2}\omega }{({L}_{zz}^{\omega })}^{{2}}{\chi }_{33}\sin \theta {\cos }^{{2}}\alpha +\frac{1}{2}{L}_{yy}^{{2}\omega }{L}_{yy}^{\omega }{L}_{zz}^{\omega }{\chi }_{15}\cos \theta \sin {2}\alpha ]}^{{2}}\\ {S}_{\parallel }\propto {|{\vec{e}}_{2\omega }^{\text{'}}\cdot {\overleftrightarrow{\chi }}_{lab}^{(2)}:{\vec{e}}_{\omega }^{\text{'}}{\vec{e}}_{\omega }^{\text{'}}|}^{{2}}\\ \quad =\,{[{L}_{zz}^{{2}\omega }{({L}_{yy}^{\omega })}^{{2}}{\chi }_{31}\cos \theta {\sin }^{{2}}\alpha +{L}_{zz}^{{2}\omega }{({L}_{zz}^{\omega })}^{{2}}{\chi }_{33}\cos \theta {\cos }^{{2}}\alpha -\frac{1}{2}{L}_{zz}^{{2}\omega }{L}_{yy}^{\omega }{L}_{zz}^{\omega }{\chi }_{15}\sin \theta \sin {2}\alpha ]}^{{2}}\end{array},$$

where ${\mathop{{e}}\limits^{{\rightharpoonup }}}_{{\Omega }}^{{\text{'}}}{=}{\mathop{{L}}\limits^{{\rightharpoonup }}}_{{\Omega }}\cdot {\mathop{{e}}\limits^{{\rightharpoonup }}}_{{\Omega }}$ denotes for the unit polarization vector of electric field with light frequency *Ω*, $\mathop{{L}}\limits^{{\rightharpoonup }}$ stands for the transmission Fresnel factor^5^, and *α* denotes for the angle between directions of the incident light polarization and the *y-*axis, as shown in Fig. 1.
